# Laryngeal Carcinoma Characteristics Associated with Positive Margins and Endoscopic Understaging

**DOI:** 10.3390/diagnostics15020150

**Published:** 2025-01-10

**Authors:** Nia Labaš, Andro Košec, Mirta Peček, Tomislav Gregurić, Siniša Stevanović

**Affiliations:** 1 Department of Otorhinolaryngology and Head and Neck Surgery, University Hospital Center Sestre Milosrdnice, 10000 Zagreb, Croatia; 2 School of Medicine, University of Zagreb, 10000 Zagreb, Croatia; 3 Department of Clinical and Interventional Radiology, University Hospital Center Sestre Milosrdnice, 10000 Zagreb, Croatia

**Keywords:** laryngeal carcinoma, narrow band imaging, understaging, margins

## Abstract

**Background/Objectives**: The study aims to analyse the factors associated with positive margins and endoscopic understaging in laryngeal carcinoma. It also aims to assess the diagnostic accuracy of Narrow Band Imaging (NBI) in comparison to White Light Endoscopy (WLE) and other diagnostic methods. **Methods**: In this retrospective comparative cohort analysis, 206 patients who underwent endoscopic laser surgery for T1 and T2a glottic squamous cell carcinoma between 1 January 2016 and 30 April 2023 were included. The data were collected from endoscopy, CT, histopathology, and NBI images. Statistical analysis was performed and associations between variables were analysed using binary logistic regression and receiver operating characteristic analysis. **Results**: The types of cordectomy performed included type III (51 patients), type IV (40 patients), and type VI (23 patients). Positive margins were found in 14.01% of patients, with significant correlations observed between positive margins and bilateral laryngeal carcinoma, right-sided laryngeal carcinoma, higher clinical and histopathologic T categories, and higher NBI grade. Endoscopic understaging versus histopathologic T category correlated with various factors, including cordectomy type, tumour size, and clinical T category. The NBI findings correlated with positive margins but did not correlate with endoscopic understaging. **Conclusions**: The study highlights several clinical and pathological factors associated with positive margins and endoscopic understaging in laryngeal carcinoma. NBI demonstrated high diagnostic accuracy, correlating with histopathological results and serving as an independent predictive factor for positive margins. Recognizing these factors is crucial for improving preoperative assessments, refining treatment strategies, and enhancing patient care.

## 1. Introduction

In the United States, head and neck cancers account for around 3.7% of all malignancies and 2.4% of all cancer-related deaths in both sexes [1]. With a global incidence of over 170,000 cases per year and about 95,000 deaths in 2018, laryngeal squamous cell carcinoma (SCC) is one of the most frequent types of head and neck cancer [2].

The 5-year overall survival has not improved considerably during the previous 20 years, despite recent improvements in therapy [1,3,4,5]. Early identification, histological diagnosis, and therapy dramatically improve prognosis, lowering patient mortality and morbidity [6]. The primary objective of surgical oncology is to achieve tumour resection with clear, disease-free margins. Several studies indicate that white light endoscopy (WLE) may lead to diagnostic uncertainty regarding the histologic nature of early, superficial neoplastic lesions (such as carcinoma in situ, T1, or T2) or preneoplastic lesions like severe dysplasia [7,8].

Proangiogenic factors in laryngeal SCC allow the development of new blood vessels that supply the tumour’s metabolic requirement [9]. The histological architecture and structural anatomy of pre-existing vessels, which may be seen by nasoendoscopy utilizing WLE at later stages, are absent from these nascent vessels [10]. To enhance outcomes, early identification of laryngeal cancer is crucial for earlier detection of this alteration [11].

An optical imaging technique termed narrowband imaging (NBI) uses optical interference filters to spectrally reduce the bandwidth of light. The two wavelengths emitted from this filter are blue light, with a peak at 400–430 nm, and green light, with a peak at 515–555 nm, which are in line with the haemoglobin absorption peaks [12]. The mucosal microvasculature, also known as intraepithelial papillary capillary loops (IPCLs), is enhanced and contrasted more effectively as a result [13].

The importance of NBI in the early identification of neoplasia has been established, and it may be used to identify lesions in the oesophagus, pharynx, and oral mucosa by analysing changes in the morphology and architecture of IPCLs [14,15,16,17].

To distinguish between benign and malignant laryngeal lesions, Ni and colleagues [18] created a categorization system in 2011. It is based on the morphological alterations of laryngeal IPCLs. This system briefly explains five morphological change patterns in the IPCL. Types I through IV are frequently linked to benign or even preneoplastic transformation. Type V, which is further classified into Va, Vb, and Vc, is connected to neoplastic transformation [18]. Numerous authors advise routinely using NBI as part of the assessment and workup of laryngeal lesions since prior research has shown it to be more sensitive than WLE [19]. Transoral surgery is a minimally invasive alternative to conventional surgery and has become a primary strategy for treating laryngopharyngeal cancer. An increasing number of institutions, alongside the use of advanced diagnostic methods, are also adopting minimally invasive surgical approaches such as transoral robotic surgery [20].

Insufficient resection margins in head and neck squamous cell carcinoma surgery frequently necessitate supplementary treatments, such as re-resection or adjuvant radiotherapy with or without chemotherapy, leading to higher morbidity and a less favorable prognosis. However, excessively wide resections to achieve clear margins may also result in unnecessarily increased morbidity [21].

The objective of this study was to assess the effectiveness of an additional diagnostic modality, such as Narrow Band Imaging (NBI), in improving risk assessment and outcome prediction.

## 2. Methods

In this retrospective comparative cohort analysis, 206 patients who underwent endoscopic laser surgery for T1 and T2a glottic squamous cell carcinoma were included. The study was compiled according to STROBE recommendations. The Helsinki Declaration Revision of 1989 was followed, and it was authorised by the Institutional Review Board (No. 251-29-11-20-01-14). Patients who underwent endoscopic laser resection of early laryngeal carcinoma (T1a, T1b, and T2a glottic carcinoma) between 1 January 2016, and 30 April 2023, and whose full operative records, preoperative CT scans, and NBI images were accessible for analysis, with written informed consent obtained, met the inclusion criteria. Tumour tissue that reached the specimen’s border or was more than one millimetre from the closest free margin was deemed to have positive resection margins. All patients without invasive carcinoma were excluded from the study.

Endoscopic and CT data regarding clinical T category, NBI grade, supra/subglottic or anterior commissure involvement, tumour grade, and size were the main predictive variables, with additional factors acting as covariates. Based on the histopathologic results, cases were then divided into T1a, T1b, and T2a. The primary objective of the data analysis was to examine the tumour extension information obtained from a single patient cohort from endoscopy, CT, histopathology, and NBI, four different diagnostic sources.

**Figure 1:**
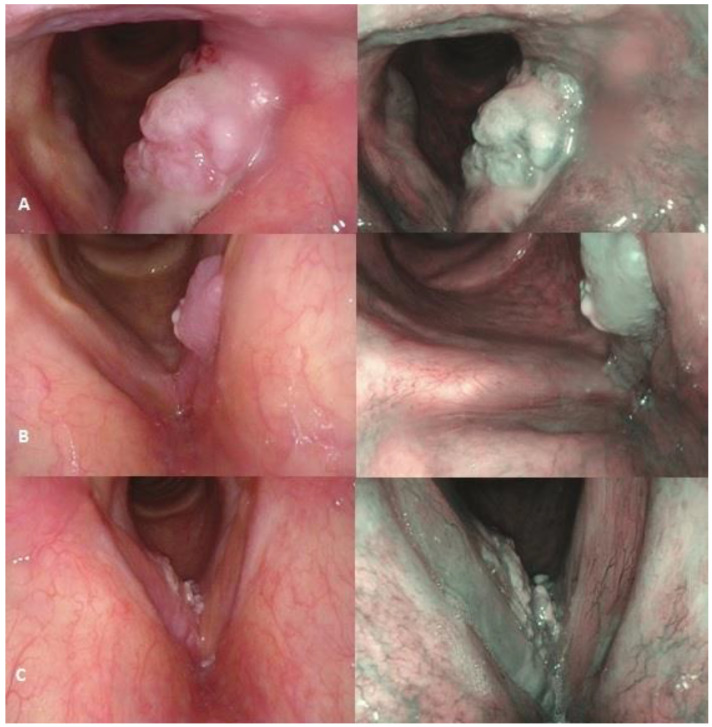
Comparison of WLE and NBI images. (**A**) Left vocal cord tumour, comparison between WL and NBI, Ni Classification V. (**B**) Left vocal cord tumour, comparison between WL and NBI, Ni Classification III. (**C**) Right vocal cord tumour expanding to the subglottical region, anterior commissure, and the left vocal cord. Comparison between WL and NBI. Ni classification V.

A preoperative phoniatric examination included nasoendoscopy with lidocaine spray used topically to anesthetize the nasal cavity. Patients were awake and seated during nasoendoscopy using a transnasal flexible fiberscope. On WLE, any vocal cord lesions that appeared alarming were noted, including leukoerythroplakia, polypoid lesions, ulcerated lesions, exophytic, and endophytic lesions. The endoscopic assessment was performed prior to the transoral laser microsurgery (see Figure 1 as example).

The lesions were then evaluated in real-time and recorded with the light in NBI mode. The corresponding NBI images obtained during nasoendoscopy were rated separately by the Ni classification after the histopathological results. Each endoscopic finding and clinically suspicious patient underwent an NBI endoscopic examination (see Figure 1 as example). Patients classified as NI V were considered highly suspicious for tumour presence. Then, the relevant Ni classification was assigned to postoperative histopathological grades. The gold standards for diagnosis were histopathological results. All patients were examined and staged preoperatively and were evaluated by a single experienced endoscopist, with the same approach applied for CT scans by an experienced radiologist and histopathological analysis by a single experienced pathologist. ENT specialists, according to ELS guidelines, conducted regular follow-ups: every 2 months during the first 2 years, every 3 months in the 3rd year, and every 6 months in the 4th and 5th years. [22]. All patients with endoscopic and CT features indicative for minimal surgery candidacy were treated with transoral endoscopic surgery, with a second-look surgery performed in select cases 6 weeks after the primary surgery. A repeated biopsy was conducted if any adverse characteristics of the postoperative site were noted after primary surgery with negative margins. The main criterium for RT was positive margins after primary surgery or positive histopathology after second-look surgery.

SPSS software (version 22.0; IBM) was used to conduct statistical analysis. An odds ratio (OR)-based binary logistic regression model was used to analyse associations between variables. With the use of receiver operating characteristic analysis, correlations were further examined. Statistical significance was defined as an area under the curve > 0.6. A two-sided 5% type I error rate was used for all tests.

## 3. Results

The mean ± SD age of patients was 65. The oldest patient was 90 and the youngest 36 years old, with a male to female ratio of 192:14.

Surgery or radiotherapy treatment was not performed in any of the patients prior to examination. The type of surgery was labelled according to the ELS cordectomy classification, with cordectomy type III performed in 51, cordectomy type IV in 40, and cordectomy type VI performed in 23 patients.

In the patient cohort, 81 (39.32%) patients had squamous cell carcinoma limited to the left vocal cord, 99 (48.05%) patients had squamous cell carcinoma on the right vocal cord, and 25 (12.14%) patients showed bilateral spread of the disease. There were 29 (14.01%) patients with positive margins. The intraoperative endoscopic finding differed from CT staging in 71 patients (34.95%) and from the definitive histopathologic findings in 77 (37.38%) patients (Table 1).

The binary logistic regression model that tested the dependent variable of the occurrence of positive margins (Table 2) identified a positive correlation with bilateral laryngeal carcinoma (*p* = 0.016; OR, 8.284), right-sided laryngeal carcinoma (*p* = 0.004; OR, 8.176), rising clinical T category (*p* = 0.042; OR, 6.324), rising histopathologic T category (*p* = 0.004; OR, 11.246), and rising NBI grade (*p* = 0.22; OR, 5.248).

Occurrence of endoscopic understaging versus histopathologic T category (Table 3) correlated with cordectomy type III (*p* = 0.045; OR, 8.051), cordectomy type V (*p* = 0.008; OR, 6.967), presence of tumour understaging on CT versus endoscopy (*p* = 0.000; OR, 12.430), clinical T1a category (*p* = 0.000; OR, 43.555), clinical T1b category (*p* = 0.000; OR, 37.417), histopathological T1a staging (*p* = 0.013; OR, 8.714), T1b staging (*p* = 0.041; OR, 4.161),the size of the tumour in centimetres (*p* = 0.001; OR, 10.949), and carcinoma recurrence (*p* = 0.018; OR, 5.560). The NBI endoscopic findings (Ni classification) did not correlate with endoscopic understaging versus histopathological T category.

The occurrence of CT over staging versus endoscopic findings correlated with the presence of right sided laryngeal carcinoma (*p* = 0.036, OR 25.99), histopathologic T category (*p* = 0.041, OR 51), and showed a negative correlation with tumour size in centimetres (*p* = 0.008, OR 0.017), postoperative radiotherapy (*p* = 0.001, OR 0.001), and with the presence of endoscopic and histopathologic finding mismatch (*p* = 0.005, OR 0.014) (Table 4).

## 4. Discussion

This study provides insights into factors associated with the occurrence of positive margins and endoscopic understaging in the context of laryngeal carcinoma, offering valuable information for understanding and managing these cases. At present, the diagnosis of laryngeal carcinoma mainly depends on the histopathological results of a biopsy or total excision. As the incidence of laryngeal carcinoma continues to rise, there is a growing necessity for improved diagnostic methods both prior to and during surgery. Ahmadzada et al. demonstrated that NBI is highly effective in diagnosing laryngeal carcinoma, achieving a sensitivity of 95.0% and a specificity of 83.3% in their study with 56 patients [11]. These results are consistent with our findings, reinforcing the diagnostic accuracy of NBI for laryngeal carcinoma. Our results indicate that the sensitivity of Ni index in relation to po positive margins was 27.6%, while specificity was 68.2%. When comparing Ni status and disease recurrence, sensitivity was 47.8% and specificity was 67.8%. The published literature indicates that routine use of CT scans for every patient in early laryngeal cancer stages is not necessary, as CT imaging was found to have limited accuracy for superficial laryngeal lesions and T1a tumours. The potential for the tumour mass within the larynx to distort surrounding structures on CT imaging can result in tumour overstaging, as observed in our findings, and may discourage attempts at endoscopic treatment (Table 1) [23].

The occurrence of positive margins holds particular significance after transoral CO_2_ laser microsurgery. Hendriksma et al. reported on 84 patients who were staged by endoscopy and had biopsy-proven SCC, revealing that only 19% of patients exhibited negative margins and 14 patients with positive margins (20.6%) developed local recurrence [24]. Studies examining the role of positive margins present considerable variations in their recommendations [25,26]. Klimza et al. examined the histological examination of margins guided by NBI and White Light WL examination, revealing the impact of NBI on intraoperative decision-making. Their findings underscored the superiority of NBI over WL [27]. Also, according to Garafolo et al., pre- and intraoperative evaluations by NBI examination represent a useful diagnostic tool in comparison to patients treated without NBI. In their study, patients examined pre- and intraoperatively with NBI showed a significant reduction in positive margins (from 23.7% to 3.6%) [28]. A study investigating transoral robotic surgery reported that the intraoperative use of Narrow Band Imaging (NBI) demonstrated a sensitivity of 72.5% and a specificity of 66.7%, with a negative predictive value of 87.9%. Additionally, frozen section analysis of surgical specimen margins showed a significantly higher rate of negative superficial lateral margins in cases utilizing NBI compared to those using white light [29]. NBI endoscopic findings in our study correlated with positive margins found in histopathological results, while they did not correlate with endoscopic understaging. Therefore, it serves as an independent measure indicating the presence of a tumour and is an independent predicting factor of a positive margin. Prior to the adoption of intraoperative imaging techniques like NBI, the literature reports a high incidence of positive neoplastic margins following laser microsurgery—reaching up to 40% in some series. However, pathological examinations from revision surgeries often reveal no residual disease in the majority of these cases (up to 80–90%). In a 2023 study, Kleijn et al. reported that various advanced techniques, including Narrow Band Imaging (NBI), high-resolution microendoscopic imaging, confocal laser endomicroscopy, frozen section analysis (FSA), ultrasound (US), computed tomography (CT), (auto) fluorescence imaging (FI), and augmented reality (AR), have been utilised in the management of oropharyngeal carcinomas. Among these, NBI, FSA, and US are the most commonly employed, significantly contributing to higher rates of negative surgical margins [30]. The study from Dobashi et al. suggests that Texture and Color Enhancement Imaging (TXI) may prove to be more effective than both White Light Imaging (WLI) and Narrow-Band Imaging (NBI) in enhancing the visibility of squamous cell carcinoma (SCC) suspicious lesions in the pharynx and esophagus [31]. However, we could not find studies that examine positive margins in laryngeal carcinomas and their association with TXI.

The constraints inherent in our study encompassed a limited sample size and a relatively brief duration of follow-up. A limitation of our study was the inability to directly link the variable to survival outcomes. Instead, the closest possible variable associated with survival was used—positive margins, as confirmed by pathohistological analysis. Additionally, the limitations of our study include the fact that NBI was used exclusively for preoperative evaluation of patients and not for intraoperative purposes.

The strengths of the study include the relatively large retrospective cohort of patients with early laryngeal cancer that were treated at this centre as well as the broad use of NBI in the diagnostic work-up of these patients.

## 5. Conclusions

In summary, the results highlight various clinical and pathological factors that are linked to the occurrence of endoscopic understaging in cases of laryngeal carcinoma. Further studies should either validate or prospectively study the utility of CT for early T lesions of the larynx, since the data suggest that CT imaging has limitations in assessing early-stage laryngeal carcinoma. In contrast, the incorporation of NBI diagnostics, along with the Ni classification, which has not yet been introduced as a routine diagnostic method, aids in risk assessment and prognosis. This study provides valuable insights into the role of Narrow Band Imaging (NBI) in the early detection and intraoperative management of early glottic carcinomas, aiming to reduce the incidence of postoperative positive margins. NBI has proven to be a useful tool for better delineating tumour borders during transoral laser microsurgery (TLM), significantly decreasing the rate of positive superficial margins. The management of positive or close resection margins following TLM remains a complex and debated issue.

## Figures and Tables

**Table 1:** Patient characteristics (by gender, type of surgery, endoscopic, CT and histopathological T staging). Data are presented as count (%) or mean (standard deviation).

Characteristics	All Patients
N	206
Mean age (years)	65 (±10)
Female/Male	14/192
Endoscopic T staging (WLI and NBI)	
T1a	146
T1b	39
T2a	21
Type of surgery	
Type III cordectomy	51 (24.76%)
Type IV cordectomy	40 (19.42%)
Type Vabcd cordectomy	92 (44.66%)
Type VI cordectomy	23 (11.17%)
Histopathologic T staging	
T1a	146
T1b	42
T2a	18
Mismatch of endoscopic and CT staging	71 (34.95%)
Clinical overstaging	36 (50.70%)
Clinical understaging	35 (49.29%)
Mismatch of endoscopic and histopathologic staging	77 (37.38%)
Clinical overstaging	33 (42.86%)
Clinical understaging	39 (50.65%)
Positive postoperative margins	29 (14.08%)

**Table 2:** Positive predictors for positive margins identified with binary logistic regression (*p <* 0.05, OR—odds ratio).

Positive margins as the outcome variable	
Predictor variable	*p* value (OR)
Bilateral laryngeal carcinoma	0.016 (8.284)
Right-sided laryngeal carcinoma	0.004 (8.176)
Rising clinical T category	0.042 (6.324)
Rising histopathologic T category	0.004 (11.246)
Rising NBI grade	0.022 (5.248)

**Table 3:** Positive predictors for endoscopic and histopathologic staging discrepancy identified with binary logistic regression (*p <* 0.05, OR—odds ratio).

Endoscopic and histopathologic discrepancy as the outcome variable	
Predictor variable	*p* value (OR)
Cordectomy type III	0.045 (8.051)
Cordectomy type V	0.008 (6.967)
Clinical T1a	0.000 (43.55)
Clinical T1b	0.041 (4.161)
Size of tumour (cm)	0.001 (10.949)
Carcinoma recurrence	0.018 (5.560)

**Table 4:** Positive predictors for CT overstaging and endoscopic findings discrepancy identified with binary logistic regression (*p <* 0.05, OR—odds ratio).

CT and endoscopic findings as the outcome variable	
Predictor variable	*p* value (OR)
Right sided laryngeal carcinoma	0.036 (25.99)
Histopathologic T category	0.041 (51)
Tumour size (cm)	0.008 (0.017)
Postoperative radiotherapy	0.001 (0.001)

## Data Availability

The original contributions presented in this study are included in the article. Further inquiries can be directed to the corresponding author. The raw data supporting the conclusions of this article can be made available by the authors on request.
